# Persisting exercise ventilatory inefficiency in subjects recovering from COVID-19. Longitudinal data analysis 34 months post-discharge

**DOI:** 10.1186/s12890-024-03070-1

**Published:** 2024-05-25

**Authors:** Gianluigi Dorelli, Giulia Sartori, Giulia Fasoli, Nicolò Ridella, Nicola Bianchini, Michele Braggio, Marcello Ferrari, Massimo Venturelli, Luca Dalle Carbonare, Carlo Capelli, Bruno Grassi, Ernesto Crisafulli

**Affiliations:** 1 School of Medicine in Sports and Exercise, University of Verona, Verona, Italy; 2 Department of Medicine, Respiratory Medicine Unit, University of Verona and Azienda Ospedaliera Universitaria Integrata of Verona, Largo L. A. Scuro, 10, Verona, 37124 Italy; 3 Department of Neuroscience, Biomedicine and Movement Sciences, University of Verona, Verona, Italy; 4 Department of Medicine, University of Udine, Udine, Italy

**Keywords:** COVID-19, Cardiopulmonary exercise test, Exercise ventilatory inefficiency, Hyperventilation, End-tidal pressure of CO_2_, Oxygen pulse

## Abstract

**Background:**

SARS-CoV-2 infection has raised concerns about long-term health repercussions. Exercise ventilatory inefficiency (EV*in*) has emerged as a notable long-term sequela, potentially impacting respiratory and cardiovascular health. This study aims to assess the long-term presence of EV*in* after 34 months and its association with cardiorespiratory health in post-COVID patients.

**Methods:**

In a longitudinal study on 32 selected post-COVID subjects, we performed two cardiopulmonary exercise tests (CPETs) at 6 months (T0) and 34 months (T1) after hospital discharge. The study sought to explore the long-term persistence of EV*in* and its correlation with respiratory and cardiovascular responses during exercise. Measurements included also V̇O_2peak,_ end-tidal pressure of CO_2_ (PET_CO2_) levels, oxygen uptake efficiency slope (OUES) and other cardiorespiratory parameters, with statistical significance set at *p* < 0.05. The presence of EV*in* at both T0 and T1 defines a persisting EV*in* (pEV*in*).

**Results:**

Out of the cohort, five subjects (16%) have pEV*in* at 34 months. Subjects with pEV*in*, compared to those with ventilatory efficiency (Ev*ef*) have lower values of PET_CO2_ throughout exercise, showing hyperventilation. Ev*ef* subjects demonstrated selective improvements in DL_CO_ and oxygen pulse, suggesting a recovery in cardiorespiratory function over time. In contrast, those with pEv*in* did not exhibit these improvements. Notably, significant correlations were found between hyperventilation (measured by PET_CO2_), oxygen pulse and OUES, indicating the potential prognostic value of OUES and Ev*in* in post-COVID follow-ups.

**Conclusions:**

The study highlights the clinical importance of long-term follow-up for post-COVID patients, as a significant group exhibit persistent EV*in*, which correlates with altered and potentially unfavorable cardiovascular responses to exercise. These findings advocate for the continued investigation into the long-term health impacts of COVID-19, especially regarding persistent ventilatory inefficiencies and their implications on patient health outcomes.

**Supplementary Information:**

The online version contains supplementary material available at 10.1186/s12890-024-03070-1.

## Introduction

Post-COVID condition refers to a range of symptoms and clinical findings that persist following the acute phase of SARS-CoV-2 infection [1]. In these patients, the cardiopulmonary exercise test (CPET) has highlighted a reduction of maximal exercise capacity and oxygen uptake (V̇O_2peak_) and has been helpful in elucidating the underlying pathophysiological mechanisms leading to exercise intolerance and unexplained perceived dyspnea [1, 2]. CPET has demonstrated that exercise hyperventilation and ventilatory inefficiency (Ev*in*) are a contributor to numerous disabling signs and symptoms in post-COVID patients, such as persisting breathlessness and long-lasting exercise intolerance [3, 4].

Exercise ventilation efficiency is assessed by examining how minute ventilation (V̇E) correlates with the amount of carbon dioxide produced (V̇CO_2_). This relationship is quantified using three metrics: the slope of V̇E against V̇CO_2_ (V̇E/V̇CO_2slope_), the lowest value observed (nadir) for this ratio, and the carbon dioxide ventilatory equivalent at the first ventilatory threshold (V̇E/V̇CO_2_ at θL) [5]. These metrics are well-established for evaluating mismatches in ventilation and pulmonary perfusion during exercise in patients with heart and lung conditions [6]. High values of V̇E/V̇CO_2_ relationship commonly indicate EV*in*, which is a condition of breathing dysfunction related to excessive ventilation [5].

Ventilatory inefficiency is a global indicator of cardiorespiratory response to exercise and a well-recognized prognostic marker in chronic patients second only to V̇O_2peak_ [7]. As pointed out by Weatherald et al., EV*in* is also a hallmark of pulmonary vascular diseases, such as pulmonary arterial hypertension and chronic thromboembolic pulmonary hypertension where it is an excellent prognostic marker [8].

Understanding the pathophysiological origins of EV*in* is essential to comprehending the exercise response in post-COVID syndrome. A significant amount of evidence indicates that a subset of asymptomatic COVID-19 survivors exhibits EV*in*, with prevalences reported at 29% and 17% at 6 and 12 months post-discharge, respectively [9–11]. Compared to those without exercise ventilatory inefficiency, those with ventilatory efficiency (Ev*ef*), post-COVID patients with Ev*in* show lower values of end-tidal pressure of CO_2_ (PET_CO2_) throughout the exercise and display hypocapnia and respiratory alkalosis, which may correlate with an impairment in diffusing capacity (DL_CO_) [3, 4, 10, 12].

Moreover, evidence at 12 months following severe COVID-19 infections indicates that numerous patients, despite achieving normal V̇O_2peak_ levels, exhibit signs of Ev*in*, notably linked to signs of underlying pulmonary microvascular disease and increased dead space ventilation [13]. Such vascular complications are believed to stem from endothelial dysfunction and a hypercoagulable state, both of which are acute sequelae of the systemic inflammatory response to SARS-CoV-2 infection [13].

An invasive CPET study documents that symptomatic long-COVID patients with reduced exercise capacity have a blunted peripheral oxygen extraction [14]. However, in asymptomatic patients, exercise limitations are less clear and still need to be clarified. In addition to V̇O_2peak_ and V̇_E_/V̇_CO2_ relationship, impairments of the respiratory and cardiovascular response to exercise, could be also evaluated through the oxygen pulse (O_2_ pulse), aerobic efficiency slope (V̇_O2_/W_slope_) and oxygen uptake efficiency slope (OUES) which also estimates the cardiovascular risk in certain populations [15, 16]. O_2_ pulse is the ratio between oxygen uptake and heart rate (HR): it reflects the amount of oxygen extracted by the tissue per heartbeat and could be used as a non-invasive estimator of stroke volume, or peripheral oxygen utilization [7].

Despite these parameters being less strong indicators for evaluating overall survival in the general population, some recent long-term longitudinal studies show that low O_2_ pulse at peak and OUES have been associated with increased cardiovascular and all-cause mortality in certain populations [15–17] These data need to be further confirmed by other similar longitudinal studies: however, evidence shows that post-COVID patients have a reduced aerobic capacity and O_2_ pulse independent from V̇O_2peak_ levels [18]. While this data could not be interpreted in terms of long-term implications, they could be a subclinical sign of altered cardiovascular response due to the infection in these patients [19].

The enduring clinical significance of EV*in* and the altered cardiovascular response to exercise in post-COVID patients remains an area of ongoing investigation [13, 20]. The persistence of these conditions after 1 year following hospital discharge underscores the need for pathophysiological investigations and sustained longitudinal studies.

Our study aims to explore the persistence of EV*in* in post-COVID patients and to unravel its potential long-term repercussions on respiratory and cardiovascular health.

Our first hypothesis is that EV*in* may persist chronically after COVID-19 infection. Evidence suggests that it could be a sign of acute SARS-CoV-2 infection and a subclinical impairment of exercise response which involves both the cardiovascular and the respiratory systems and this leads to our second hypothesis. We also hypothesized that EV*in* is a sign of a broader dysfunction in the cardiorespiratory response, which may also correlate with signs of an increased cardiovascular risk.

## Methods

### Selection of patients

**Fig. 1:**
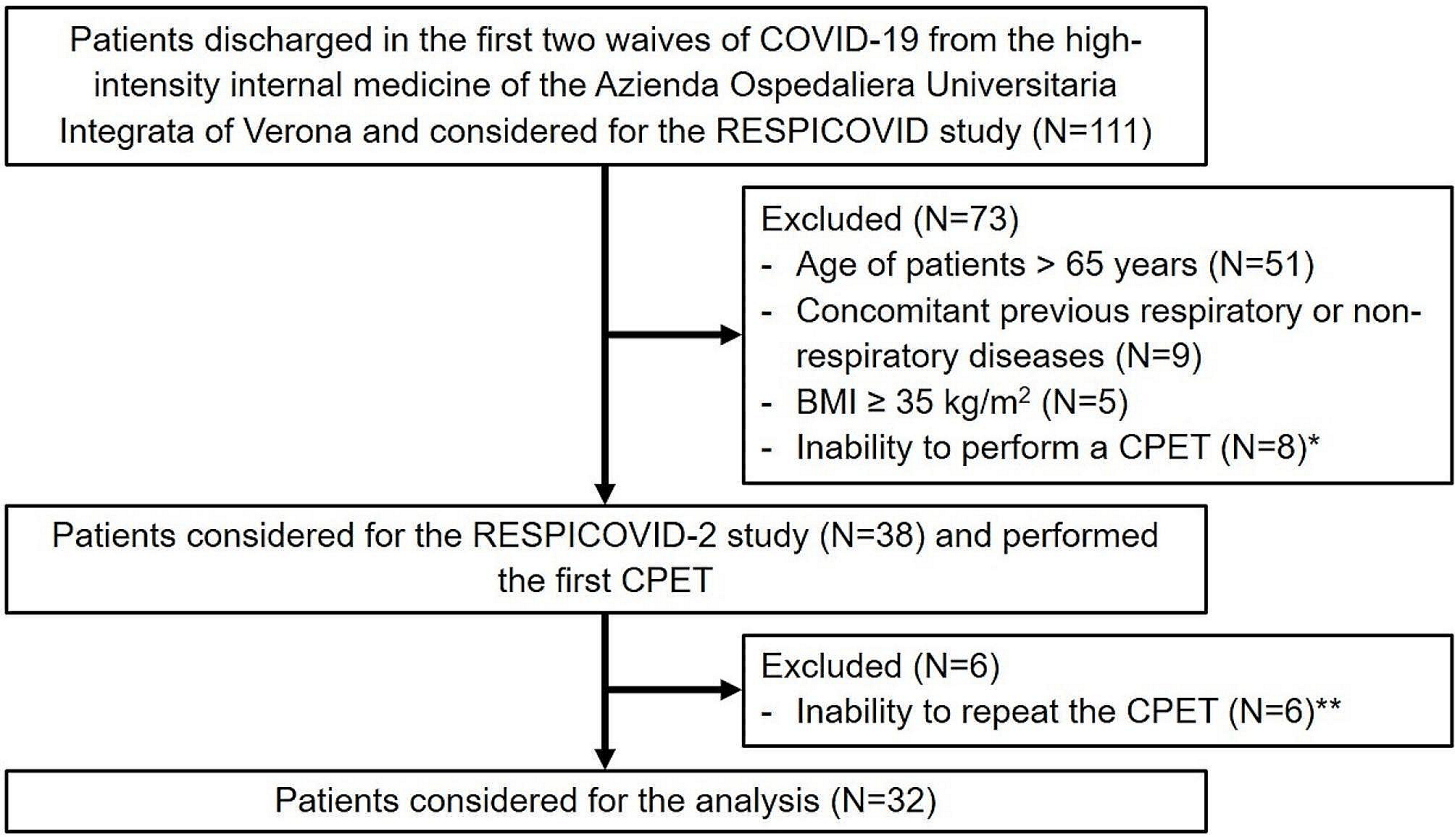
Study flow diagram *Abbreviations*: BMI defines body mass index; CPET, cardiopulmonary exercise test. *Patient were not able to perform maximal CPET due to musculoskeletal symptoms. **Patients were excluded due to personal unavailability, refusal to continue with the study, or the emergence of new musculoskeletal conditions that limited their ability to exercise

We evaluated the resting and exercise ventilatory and cardiovascular responses in a cohort of selected post-COVID patients at 34 months from hospitalization, comparing data with a previous evaluation performed 6 months after discharge. Data were collected from the RESPICOVID initiative, a prospective observational study conducted at the Respiratory Medicine Unit of the University of Verona and Azienda Ospedaliera Universitaria Integrata of Verona (Italy), involving patients hospitalized for COVID-19 pneumonia during the first two waves of the pandemic emergency in Italy. A dedicated outpatient clinic has been organized, and all subjects discharged were considered. The present longitudinal analysis with repeated measures has been designed to evaluate the long-term persistence of ventilatory inefficiency in subjects enrolled in the RESPICOVID-2 study [11]. Only subjects who performed both CPETs (at T0 and T1) were considered. Figure 1 shows the study flow diagram.

To better define the EV*in* and cardiovascular response to exercise, we excluded any potential physiological or pathological variable influencing exercise adaptations [6]. We have then excluded subjects meeting the following criteria: *(a)* age exceeding 65 years; *(b)* concurrent presence of respiratory and non-respiratory chronic diseases (including the suspected clinical presentation of new-onset), respiratory failure, or need for long-term oxygen therapy; *(c)* a body mass index (BMI) ≥ 35 kg/m^2^; *(d)* an inability to perform a CPET with a peak respiratory exchange ratio (RER) < 1.05 (to exclude poor motivation); and *(e)* psychiatric disorders in order to avoid psychogenic hyperventilation. Among chronic diseases, only stable systemic arterial hypertension was accepted.

### Measurements

All measures were prospectively collected beginning in July 2020, approximately 6 months after the subjects’ discharge (T0), and repeated until March 2023, 34 months after the discharge (T1). Only subjects with both CPET measures (T0 and T1) were considered for the analysis. Preliminary data about measures performed at T0 have been reported previously [11]. The local Ethics Committee approved the study protocol (no. 2785CESC), which was performed according to the Good Clinical Practice recommendations and the requirements of the Declaration of Helsinki. Written informed consent was obtained from all subjects.

### Lung function

Lung function procedures were performed according to international recommendations [21–23]. A flow-sensing spirometer connected to a computer for data analysis (Jaeger MasterScreen PFT System) was used to measure lung function. Forced vital capacity (FVC), forced expiratory volume in the first second (FEV_1_), and total lung capacity (TLC) were recorded. FEV_1_/FVC ratio was taken as the index of airflow obstruction. The single-breath method measured the diffusion capacity for carbon monoxide (DL_CO_). FEV_1_, FVC, TLC, and DL_CO_ were expressed as percentages of the predicted values [22, 23].

### Cardiopulmonary exercise test

According to the ATS/ACCP Statement, for the CPET measures, we used a cycle ergometer (E100, Cosmed Srl, Rome, Italy) with a ramp protocol of 10 to 25 watts increment every minute and based on the predicted peak power output, to achieve an exercise time between 8 and 12 min [24]. Patients were monitored 3 min before the ramp protocol (rest phase) and 5 min after (cool down phase). Subjects were asked to avoid caffeine, alcohol, cigarettes, and strenuous exercise 24 h before the day of testing and avoid eating for the 2 h before the test. Subjects suspended β-blockers before testing but could take their current antihypertensive therapies. During the test, subjects were asked to maintain a pedal frequency of 65 per minute and were continuously monitored [24]. Subjects were continuously monitored with a 12-lead electrocardiogram (ECG) and a pulse oximeter; blood pressure was measured every two minutes. Stopping criteria consisted of symptoms, such as unsustainable perceived dyspnoea or leg fatigue, chest pain, a significant ST-segment depression at ECG, or a drop in systolic blood pressure or oxygen saturation ≤ 84% [24]. Cardio-respiratory measures were sampled continuously with a breath-by-breath method using a gas analysis system (Quark CPET, Cosmed Srl, Rome, Italy). Oxygen uptake was expressed in mL/kg/min and as a percentage of predicted. The ventilatory response during exercise was through the relationship of V̇_E_ against V̇_CO2_ obtained every 10 s, excluding data above the respiratory compensation point (RCP). We gathered data of V̇_E_/V̇_CO2 slope_ and Y-intercept (V̇_E_/V̇_CO2 intercept_) values obtained from the regression function. V̇_E_/V̇_CO2_ was also been evaluated at nadir (V̇_E_/V̇_CO2 nadir_) and the first ventilatory threshold (V̇_E_/V̇_CO2_ at θ_L_) [7].

For the definition of the EV*in*, we used the regression equation of V̇_E_/ V̇_CO2 slope_ for healthy subjects [5]. Related to our small sample and to avoid false positive results, we considered three standard deviations as the upper limit to define EV*in* [5]. Then, we considered subjects having a lower range of V̇_E_/V̇_CO2 slope_ (EV*ef*) and subjects with over the upper limit of V̇_E_/V̇_CO2 slope_ (EV*in*). Subjects having EV*in* at T0 and T1 were defined as persisting ventilatory inefficiency subjects (pEV*in*).

The end-tidal pressure of CO_2_ (PET_CO2_, in mmHg) was measured as the mean of PET_CO2_ during the 3-minute rest period and the last 20 s of the test and was recorded at any time during CPET (at rest, at θ_L_, at the respiratory compensation point - RCP, and at peak of exercise).

The cardiovascular response to exercise was expressed by HR, O_2_ pulse, OUES, V̇_O2_/W_slope_ and HR after 1 min of recovery (heart rate recovery, HRR). O_2_ pulse was calculated by dividing instantaneous V̇O_2_ by HR [7]. The OUES describes the relationship between V̇_O2_ and V̇_E_ during incremental exercise, via a log transformation of V̇_E_, and was expressed in L/min as the gradient of the linear relationship of log_10_ V̇_E_ to V̇_O2_ [25]. V̇_O2_/W_slope_ was calculated as the slope of oxygen uptake as a function of Watts [7, 25]. OUES thus represents the absolute rate of increase in oxygen uptake per 10-fold increase in minute ventilation. HRR in bpm was defined as the reduction in the HR from the peak exercise level to the rate 1 min after the end of exercise [26].

At the end of the exercise, dyspnoea and leg fatigue were measured by a Borg 6–20 rate perceived exertion (RPE) scale [27]. Perceived peak dyspnoea and fatigue data have been described as RPE and peak workload ratio. We considered a test as maximal if subjects had a plateau of the V̇O_2_ for more than 20 s, a Respiratory Exchange Ratio (RER) > 1.15, and a Borg RPE score > 18 [24].

### Self-reported questionnaire

The modified Medical Research Council (mMRC) questionnaire was administered to measure perceived breathlessness, with a range from 0 (shortness of breath with strenuous exercise) to 4 (too breathless to leave the house) [28]. The Italian version of the International Physical Activity Questionnaire (IPAQ) was administered to measure the daily physical activity of the subjects estimating, the three levels of the metabolic equivalent of task (METs): inactive, minimally active, and health- enhancing physical activity (HEPA) active [29].

### Statistical analysis

A preliminary Shapiro-Wilk test was performed. Data are reported as percentages for categorical variables, as mean (SD) or median [IQR-interquartile range] for continuous variables with a normal or non-normal distribution, respectively. Categorical variables were compared using the Chi-square test or the Fisher exact test. According to the distribution of continuous variables, the independent *t*-test or the non-parametric Mann-Whitney test were used to compare EV*ef* and pEV*in* groups, while the paired *t*-test, or the non-parametric Wilcoxon signed-rank test were used to compare the differences between T1 and T0. Relationships between variables were assessed using Pearson’s correlation coefficient (*r*).

All analyses were performed using IBM SPSS, version 17.0 (IBM Corp., Armonk, NY, USA), with *p*-values of < 0.05 considered statistically significant.

## Results

We evaluated the same thirty-two post-COVID subjects at T0 (median time from discharge 184 days) and T1 (median 1015 days). At T0, of 32 subjects, 8 had EV*in* (25%), while at T1 5 subjects (16%) had a pEV*in*. Subjects with pEV*in*, in comparison to subjects with EV*ef*, had significantly higher values of a baseline of V̇E/V̇CO_2 slope_, V̇_E_/V̇_CO2 nadir_, and V̇E/V̇CO_2_ at θ_L_ with lower values of V̇E/V̇CO_2 intercept_. No other variables, including those related to COVID-19 hospitalization, differed between subjects with pEV*in* and subjects with EV*ef*. Baseline variables were reported in Table 1. Supplementary Table 1 reports the characteristics of EV*in* and pEV*in* patients.

**Table 1:** General, functional and CPET-related baseline variables

Variables	All subjects (*N* = 32)	Subjects with EV*ef* (*N* = 27)	Subjects with pEV*in* (*N* = 5)	*p*-value
Age, y	55.2 [9]	55 [5.5]	58 [13.1]	0.550
Male, *n* (%)	24 (75)	20 (74)	4 (80)	> 0.999
Current or former smokers, *n* (%)	18 (56)	15 (56)	3 (60)	> 0.999
Arterial hypertension^*^, *n* (%)	10 (31)	9 (33)	1 (20)	> 0.999
BMI, kg/m^2^	26.8 ± 3.3	26.7 ± 3.4	27.3 ± 3.2	0.734
FEV_1_, % predicted	115.7 ± 13.9	114.2 ± 13.8	126.2 ± 9.9	0.107
FVC, % predicted	119 [21]	117.5 [19]	124.5 [18]	0.376
FEV_1_/FVC, %	79.3 ± 5.8	78.8 ± 5.9	82.3 ± 5.4	0.279
TLC, % predicted	102.7 ± 11.9	103 ± 11.8	100.5 ± 13.2	0.697
DL_CO_, % predicted	92.6 ± 13.4	92.7 ± 13	91.7 ± 17.5	0.897
PaO_2_, mmHg	101.9 ± 11.8	103 ± 11.7	96.4 ± 12	0.261
PaCO_2_, mmHg	38.7 ± 3.1	38.5 ± 3.2	39.6 ± 3	0.482
pH	7.42 ± 0.03	7.43 ± 0.03	7.42 ± 0.01	0.640
6MWT, total distance walked meters	587.8 ± 84.3	592.4 ± 82.3	562.8 ± 101	0.480
mMRC, score	1 [0]	1 [0]	1 [1]	0.880
IPAQ (inactive/minimally active/HEPA active), *n* (%)	8 (25)/17(53)/7(22)	5 (19)/16(59)/6(22)	3 (60)/1(20)/1(20)	0.126
METs, total	1407 [2090]	1428 [2214]	1386 [2068]	0.815
Workload, watts	166.6 ± 50.8	169.9 ± 52.1	148.8 ± 43.4	0.401
V̇_O2_ at peak, ml	2114.9 ± 548.3	2143.6 ± 561.7	1960.2 ± 493.7	0.501
V̇_O2_ at peak, ml/kg/min	26.2 ± 5.2	27 ± 5.7	24.7 ± 7.2	0.427
V̇_O2_ at peak, % predicted	98.7 ± 15	100 ± 15.8	91.6 ± 6.1	0.255
V̇_O2_/W_slope_	9.71 ± 1.31	9.81 ± 1.23	9.18 ± 1.75	0.330
V̇_E_/V̇_CO2 slope_	28 ± 4	26.9 ± 3.3	33.9 ± 1.6	**< 0.001**
V̇_E_/V̇_CO2 nadir_	26.8 ± 2.6	26.2 ± 2.2	30.5 ± 2	**< 0.001**
V̇_E_/V̇_CO2_ at θ_L_	28.1 ± 2.7	27.6 ± 2.5	31 ± 1.7	**0.008**
V̇_E_/V̇_CO2 intercept_	2.79 ± 3.6	3.35 ± 3.4	-0.24 ± 3.6	**0.042**
V̇_E_ at rest, L/min	15.9 ± 5.9	15.6 ± 5.8	17.6 ± 7.2	0.489
V̇_E_ at peak, L/min	85 [40.2]	84.1 [36.6]	101.3 [38.6]	0.159
RR change^§^, breath/min	19 ± 7	18.5 ± 7.3	22.2 ± 4.1	0.282
RER at rest	0.86 ± 0.15	0.86 ± 0.15	0.89 ± 0.13	0.488
RER at peak	1.20 ± 0.08	1.19 ± 0.08	1.21 ± 0.05	0.487
O_2_ pulse at peak, mL/bpm	13.5 ± 3	13.5 ± 3.2	12.6 ± 2.3	0.579
OUES, L/min	1.09 ± 0.22	1.11 ± 0.21	0.98 ± 0.20	0.242
HR_max_	156.7 ± 14.3	157.3 ± 14.2	153.6 ± 13.9	0.593
HRR, beats/minute	23.8 ± 6.3	24.4 ± 6.3	20.6 ± 6	0.227
HR/V̇_O2_ slope, L^−1^	50.1 [33.8]	50.1 [31.8]	77.5 [56]	0.361
Perceived peak dyspnea^#^	17 [4]	17 [4]	17 [3.5]	0.525
Perceived peak fatigue^#^	18 [2]	18 [2]	18 [3]	> 0.999
*Variables related to COVID-19 hospitalization*				
Length of hospital stay, days	6 [5]	6.1 [5]	6 [11]	0.677
Needing of oxygen therapy, *n* (%)	22 (68)	18 (67)	4 (80)	> 0.999
Needing of ventilatory support, *n* (%)	13 (41)	11 (41)	2 (40)	> 0.999
Needing of ICU admission, *n* (%)	5 (16)	3 (11)	2 (40)	0.163
Pulmonary embolism, *n* (%)	2 (6)	2 (7)	0 (0)	> 0.999
PaO_2_/FiO_2_ at admission (*n* = 16)	305.9 ± 102.2	305.7 ± 107.2	307.9 ± 84.2	0.986
PaO_2_/FiO_2_ < 300, *n* (%)	9 (53)	8 (53)	1 (50)	> 0.999
PaCO_2_ at admission (*n* = 16)	34.2 ± 5.6	34.1 ± 4.9	35 ± 12.7	0.940

Data are shown as the number of subjects (%), means ± SD, or medians [IQR-interquartile range]. In bold are reported significant *p*-values of independent *t*-test, or the Mann-Whitney test

^*^Subjects with arterial hypertension were treated with ACE inhibitors (*N* = 6, 19%), β-blockers (*N* = 4, 12%), and Ca^2+^ antagonist (*N* = 3, 9%); ^§^Calculated as value at peak less value at rest; ^#^Described as a Borg 6–20 perceived exertion rate score

*Abbreviations*: EV*ef* defines exercise ventilatory efficiency; pEV*in*, persisting exercise ventilatory inefficiency; BMI body mass index; FEV_1_, forced expiratory volume at 1st second; FVC, forced vital capacity; TLC, total lung capacity; DL_CO_, diffusion capacity for carbon monoxide; PaO_2_, partial arterial oxygen pressure; PaCO_2_, partial pressure of arterial carbon dioxide; 6MWT, six-minute walking test; mMRC, modified Medical Research Council dyspnea score; IPAQ, international physical activity questionnaire; HEPA, health-enhancing physical activity; METs, metabolic equivalent of task; V̇_O2_, oxygen uptake; V̇_E_/V̇_CO2 slope_, the slope of V̇_E_ to carbon dioxide output-V̇_CO2_ ratio; θ_L_, the first ventilatory threshold; V̇_E_/V̇_CO2 intercept_, point of intercept of V̇_E_ to carbon dioxide output-V̇_CO2_ ratio; V̇_E_, minute ventilation; RER, respiratory exchange ratio; RR, respiratory rate; OUES, oxygen uptake efficiency slope; HRR, heart rate recovery; ICU, intensive care unit

In all subjects, comparing T1 vs. T0 (Table 2), there was an increment of BMI, DL_CO_ % predicted, V̇_O2_ at peak % predicted, and O_2_ pulse at peak, with a reduction of FEV_1_ and FVC (both % predicted), V̇E/V̇CO_2_ at θ_L_ and V̇_E_ at rest. In EV*ef*, selective changes between T1 and T0 were evident in the following variables: BMI, DL_CO_ % predicted, O_2_ pulse at peak, V̇_E_/V̇_CO2_ at θ_L_ and V̇_E_ at rest. No selective changes were evident in subjects with pEV*in*.

**Table 2:** CPET-related differences between T0 and T1

Variables	All subjects (*N* = 32)			Subjects with EV*ef* (*N* = 27)			Subjects with pEV*in* (*N* = 5)		
	T0	T1	*p*-value	Mean difference (T1-T0)	95% CI	*p*-value	Mean difference (T1-T0)	95% CI	*p*-value
BMI, kg/m^2^	26.8 ± 3.3	27.6 ± 3.6	**< 0.001**	0.97	0.43 to 1.53	**< 0.001**	0.20	-0.34 to 0.74	0.368
FEV_1_, % predicted	115.7 ± 13.9	113.1 ± 12.3	**0.023**	-2.2	-4.4 to 0.04	0.054	-5.5	-19.8 to 8.8	0.311
FVC, % predicted	119 [21]	115 [11]	**0.010**	-0.37	-8.3 to 7.6	0.925	-4	-13.9 to 5.9	0.289
FEV_1_/FVC, %	79.3 ± 5.8	79.2 ± 4.9	0.910	0.2	-1.1 to 1.5	0.774	-1.8	-3.8 to 0.25	0.069
TLC, % predicted	102.7 ± 11.9	101.7 ± 10.8	0.413	-1.2	-3.7 to 1.1	0.292	1.2	-12.3 to 14.8	0.789
DL_CO_, % predicted	92.6 ± 13.4	97.2 ± 12.1	**0.004**	5.2	2.1 to 8.4	**0.002**	1.7	-18.1 to 21.7	0.798
mMRC, score	1 [0]	1 [0]	0.705	0	-1.9 to 1.9	> 0.999	0.2	0.3 to 0.7	0.374
Workload, watts	166.6 ± 50.8	164.4 ± 44.9	0.462	-3.5	-10.6 to 3.6	0.327	4.4	-5-5 to 14.3	0.285
V̇_O2_ at peak, ml	2114.9 ± 548.3	2188.2 ± 545.2	0.068	76.3	-16.3 to 168.9	0.102	56.8	-91.9 to 205.5	0.349
V̇_O2_ at peak, ml/kg/min	26.2 ± 5.2	26.7 ± 5.9	0.333	-0.61	-1.8 to 0.5	0.287	0.24	-1.69 to 2.17	0.748
V̇_O2_ at peak, % predicted	98.7 ± 15	101.9 ± 13	**0.032**	2.88	-0.54 to 6.3	0.095	5	-0.4 to 10.4	0.062
V̇_O2_/W_slope_	9.71 ± 1.31	10.34 ± 1.42	**0.033**	0.50	-0.15 to 1.15	0.128	1.27	0.20 to 2.34	**0.030**
V̇_E_/V̇_CO2 slope_	28 ± 4	27.8 ± 3.9	0.756	-0.36	-1.77 to 1.04	0.598	0.78	-1.8 to 3.4	0.451
V̇_E_/V̇_CO2 nadir_	26.8 ± 2.6	26.3 ± 3.1	0.161	-0.51	-1.36 to 0.33	0.224	-0.52	-2.27 to 1.24	0.458
V̇_E_/V̇_CO2_ at θ_L_	28.1 ± 2.7	27.2 ± 3	**0.028**	-1.06	-1.94 to -0.18	**0.020**	0.04	-2.37 to 2.45	0.966
V̇_E_/V̇_CO2 intercept_	2.79 ± 3.6	3.26 ± 3.8	0.520	0.54	-1.1 to 2.2	0.514	0.06	-3.9 to 4	0.968
V̇_E_ at rest, L/min	15.9 ± 5.9	12.4 ± 2.8	**0.002**	-3.3	-0.98 to -5.7	**0.007**	-4.3	-11.9 to 3.3	0.191
V̇_E_ at peak, L/min	86.3 [42.8]	88.7 [39.6]	0.695	0.62	-4.90 to 6.15	0.818	-0.88	-18.4 to 16.7	0.896
RR change^§^, breath/min	19 ± 7	18.6 ± 4.9	0.688	0.42	-1.6 to 2.5	0.680	-4.94	-13.9 to 4.08	0.203
O_2_ pulse at peak, mL/bpm	13.5 ± 3	14.2 ± 3.5	**0.031**	0.80	-0.09 to 1.5	**0.027**	0.04	-1.46 to 1.54	0.943
OUES, L/min	1.09 ± 0.22	1.12 ± 0.24	0.186	0.03	-0.02 to 0.08	0.264	0.03	-0.04 to 0.11	0.331
HR_max_	156.7 ± 14.3	155.2 ± 13.5	0.397	-2.7	-6.4 to 1.01	0.147	5	-8.6 to 18.6	0.365
HRR, beats/minute	23.8 ± 6.3	25.5 ± 6.7	0.192	1.59	-1.32 to 4.5	0.272	2.2	-6 to 10.4	0.500
Perceived peak dyspnea^#^	17 [4]	17 [4]	0.182	-0.25	-1.4 to 0.8	0.640	0.2	-1.4 to 1.8	0.749
Perceived peak fatigue^#^	18 [2]	18 [2.75]	0.212	0.18	-0.6 to 1.03	0.658	0.01	-1.2 to 1.2	> 0.999

Data are shown as the number of subjects (%), means ± SD, or medians [IQR-interquartile range]. The difference between T1 and T0 are expressed as mean and confidence intervals at 95%. In bold are reported significant *p*-values of the paired *t*-test, or the Wilcoxon signed-rank test

^§^Calculated as value at peak less value at rest; ^#^Described as a Borg 6–20 perceived exertion rate score and peak workload ratio

*Abbreviations*: EV*ef* defines exercise ventilatory efficiency; pEV*in*, persisting exercise ventilatory inefficiency; BMI body mass index; FEV_1_, forced expiratory volume at 1st second; FVC, forced vital capacity; TLC, total lung capacity; DL_CO_, diffusion capacity for carbon monoxide; mMRC, modified Medical Research Council dyspnea score; V̇_O2_, oxygen uptake; V̇_E_/V̇_CO2 slope_, the slope of V̇_E_ to carbon dioxide output-V̇_CO2_ ratio; θ_L_, the first ventilatory threshold; V̇_E_/V̇_CO2 intercept_, point of intercept of V̇_E_ to carbon dioxide output-V̇_CO2_ ratio; V̇_E_, minute ventilation; RR, respiratory rate; OUES, oxygen uptake efficiency slope; HRR, heart rate recovery

**Fig. 2:**
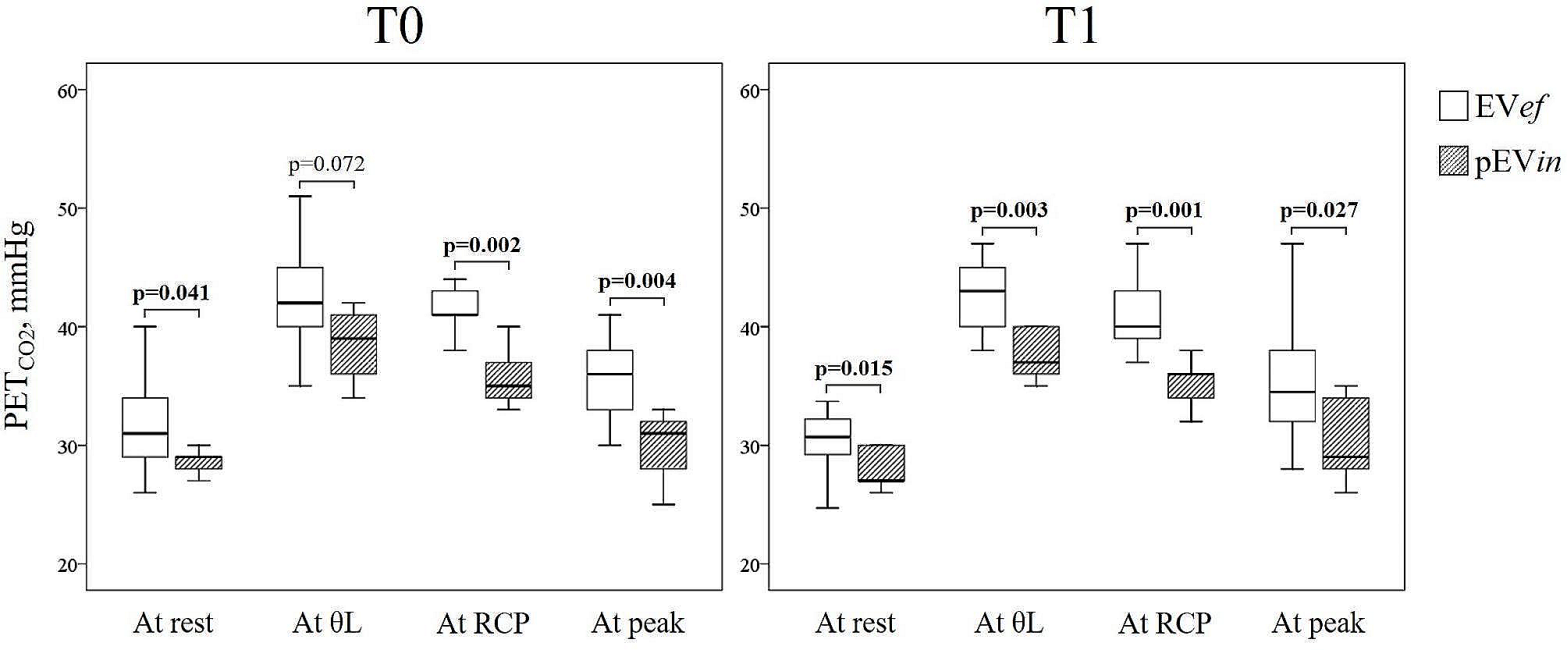
Boxplots of PET_CO2_ at any time point of CPET evaluations *Abbreviations*: EV*ef* defines the exercise ventilatory efficiency; pEV*in*, persisting exercise ventilatory inefficiency; PET_CO2_, end-tidal pressure of CO_2_; θ_L_, at the first ventilatory threshold; RCP, respiratory compensation point

PET_CO2_ was significantly lower in patients with EV*in* than EV*ef* at any time point of the exercise (at rest, at θ_L_, at RCP and peak) at T1, while at T0 were different at rest, at RCP, and peak (Fig. 2).

At T1, PET_CO2_ at rest (*r* 0.366; *p* = 0.039 and *r* 0.353; *p* = 0.048), such as at θ_L_ (*r* 0.532; *p* = 0.002 and *r* 0.586; *p* < 0.001), at RCP (*r* 0.514; *p* = 0.004 and *r* 0.565; *p* = 0.001), and peak (*r* 0.427; *p* = 0.015 and *r* 0.480; *p* = 0.005) were significantly and respectively correlated with O_2_ pulse at peak and OUES (Table 3).

**Table 3:** Correlations among variables of ventilatory inefficiency (V̇_E_/V̇_CO2 slope_), hyperventilation (PET_CO2_) and cardiovascular response to exercise (OUES, O_2_ pulse at peak), all evaluated at T1

	PET_CO2_ at rest	PET_CO2_ at θ_L_	PET_CO2_ at RCP	PET_CO2_ at peak	V̇_O2_/W_slope_	O_2_ pulse at peak	OUES
V̇_E_/V̇_CO2 slope_	*r* -0.395 *p* = 0.025	*r* -0.723 *p* < 0.001	*r* -0.801 *p* < 0.001	*r* -0.579 *p* = 0.001	*r* -0.258 *p* = 0.155	*r* -0.271 *p* = 0.134	*r* -0.339 *p* = 0.057
PET_CO2_ at rest	-	*r* 0.535 *p* = 0.002	*r* 0.432 *p* = 0.019	*r* 0.574 *p* = 0.001	*r* 0.155 *p* = 0.398	*r* 0.366 *p* = 0.039	*r* 0.353 *p* = 0.048
PET_CO2_ at θ_L_	-	-	*r* 0.941 *p* < 0.001	*r* 0.821 *p* < 0.001	*r* 0.615 *p* < 0.001	*r* 0.532 *p* = 0.002	*r* 0.586 *p* < 0.001
PET_CO2_ at RCP	-	-	-	*r* 0.815 *p* < 0.001	*r* 0.633 *p* < 0.001	*r* 0.514 *p* = 0.004	*r* 0.565 *p* = 0.001
PET_CO2_ at peak	-	-	-	-	*r* 0.538 *p* = 0.001	*r* 0.427 *p* = 0.015	*r* 0.480 *p* = 0.005
V̇_O2_/W_slope_	-	-	-	-	-	*r* 0.524 *p* = 0.002	*r* 0.632 *p* < 0.001
O_2_ pulse at peak	-	-	-	-	-	-	*r* 0.939 *p* < 0.001
OUES	-	-	-	-	-	-	-

In bold are reported significant values

Abbreviations: V̇_E_/V̇_CO2 slope_ define the slope of V̇_E_ to carbon dioxide output-V̇_CO2_ ratio; θ_L_, the first ventilatory threshold; PET_CO2_, end-tidal pressure of CO_2_; OUES, oxygen uptake efficiency slope

## Discussion

Our study starts from the hypothesis that EV*in* may be a persistent ventilo-perfusory alteration after COVID-19, which is a well-known phenomenon observed after 6 to 12 months after infection recovery [3, 4, 10, 11]. In a selected cohort of post-COVID patients, at almost three years of follow-up, we demonstrated that a pEV*in* is present in 16% of subjects. These subjects showed the phenomenon of exercise hyperventilation, documented by lower levels of PET_CO2_, and variables related to hospitalization do not seem to have a role in this alteration. However, even if not statistically significant, it seems that pEv*in* population presents a higher percentage of ICU admission (40 vs. 11%) and needs oxygen therapy, but this data will need to be confirmed by longitudinal studies with a larger sample size. Our patient cohort, comprising individuals with both EV*ef* and EV*in*, exhibited consistently normal maximal exercise capacity, as well as normal levels of FEV_1_, FVC, TLC at both 6 months (T0) and 34 months after discharge (T1). This persistent exercise hyperventilation correlates with an exacerbated cardiovascular response to exercise, which was the second hypothesis of this study.

### Ventilatory inefficiency and hyperventilation

A reduction in maximal exercise capacity and V̇_O2peak_ has been reported as the main CPET feature of symptomatic post-COVID patients [1]. However, most of the asymptomatic post-COVID patients, despite maintaining preserved lung functionality, maximal exercise capacity and V̇O_2peak_, exhibit EV*in* [10, 11]. Research has indicated that exercise ventilatory inefficiency may be a significant feature also in apparently healthy COVID-19 survivors: however, its clinical role has not yet been fully elucidated, as well as its pathophysiological cause [20].

In healthy subjects, EV*in* is uncommon and anthropometric as well as anxiety-related variables may influence it [6, 31]On the contrary, EV*in* in cardiopulmonary chronic conditions is a very common alteration and may be caused mainly by two reasons: *(1)* An altered arterial partial carbon dioxide pressure (PaCO_2_) set-point and chemosensitivity (usually a consequence of chronic hypoxemia), and *(2)* an abnormally high dead space fraction during exercise caused by a ventilatory-perfusion mismatch, which could involve the ventilation, or the pulmonary perfusion [8, 30].

Hyperventilation is a frequent manifestation of subjects recovering from COVID-19, and it is frequently associated with ventilatory inefficiency; both have been reported as a possible mechanism of persisting disabling signs and symptoms limiting exercise capacity due to an increase in the cost of ventilation [3, 4, 31]. The exact cause of this hyperventilation remains unknown. As a consequence of SARS-CoV-2 infection, an imbalance in the ventilatory control has been hypothesized as a mechanism, related to either heightened activation of activator systems (including automatic and cortical ventilatory control, peripheral afferents, and sensory cortex) or suppression of inhibitory systems (endorphins) [3]. In COVID-19 survivors, there is also a close relationship between hypocapnia resulting from resting hyperventilation and residual DL_CO_, which are the most common functional abnormalities in the early convalescence phase [12, 32]. Compared with non-severe cases, patients with severe COVID-19 had a higher impairment in DL_CO_, which likely indicates a restrictive pattern and a decrease in TLC [32]. Although the ventilatory response was unrelated to disease severity, in survival cohorts, higher values of V̇_E_/V̇_CO2 slope_ have been found in a follow-up of seven months as a predictor in developing pulmonary fibrosis [33]. Our study reports a close association between exercise hyperventilation and EV*in* as a permanent and distinctive sign of a proportion of asymptomatic survivors after 34 months (Fig. 2). Even if this phenomenon has been documented, the pathophysiological mechanism is still unclear. The clinical significance of hyperventilation and Ev*in* in cardiorespiratory conditions may be related to a perpetual altered PaCO_2_ set-point, chemosensitivity and dysautonomia [3, 4]. This reason may explain Ev*in* in asymptomatic post-COVID subjects without signs of clinical impairment and maintained exercise capacity, as described in our cohort. Most reports fail to demonstrate that this factor is independent of cardiorespiratory and endothelial damage, which led to an alteration of the ventilatory-perfusion mismatch. Some papers demonstrate a relationship between Ev*in* and residual lung function impairment in DL_CO_, especially in symptomatic long-COVID subjects [6, 12, 30].However, other papers fail to demonstrate a correlation between DL_CO_, Ev*in*, hyperventilation and a clear ventilo-perfusory mismatch [10, 11, 20]. We previously demonstrated the association between a DL_CO_ impairment and ventilatory inefficiency in post-COVID patients [11]. Now, we document a selective improvement of diffusion capacity only in EV*ef* subjects (Table 2), compared to pEv*in* subjects. Even if our study was not designed to explain the physiopathology of pEv*in*, the DL_CO_ behavior of our pEV*in* subjects after 3 years is a novel finding that merit notice as an indirect sign of subclinical damage of the cardio-respiratory system leading to an increase of dead space ventilation during exercise [2].

In line with the assessments made in a shorter period after one year of discharge, the EV*in* prevalence in our survivors (16%) is similar to that described by Ingul CB and colleagues (17%), with similar considerations about hyperventilation (PET_CO2_) [10]. Of note, Ingul CB and colleagues found a close relationship between the perceived dyspnea and EV*in*: this relationship is not confirmed in our asymptomatic patients’ cohort, in which the level of dyspnea is very low (median mMRC 1) [10]. While perceived dyspnea is typically multifaceted in nature, our methodology, which involved the selection of subjects without comorbidities and variables that might affect the ventilatory efficiency—like a subject’s weight, or a history of anxiety-related breathlessness — could have impacted these findings [6, 34]. For instance, Ingul’s study included a cohort with 29% obese patients, in contrast to our study, which comprised only three out of 32 subjects (approximately 9%) being obese (data not shown) [10]. Persistent viral presence, long-term inflammation, microclots, and hypoxia may contribute to developing symptoms in obese subjects [35]. Moreover, obesity, related to the alteration of mechanical lung function, may affect the subject’s dyspnoea perception a priori [36].

### Cardiovascular response to exercise in patients with pEVin

COVID patients are at risk for cardiovascular disease during the acute phase of the infection [19]. Due to the damage of pulmonary endothelium and microclots during the disease, we cannot exclude long-term cardiovascular complications in these patients. EV*in* is a well-recognized hallmark of pulmonary vascular disease and increased dead-space ventilation [30]. Despite normal V̇O_2peak_ levels in subjects recovered from severe COVID after one year of follow-up, dead space ventilation correlates with D-Dimer plasma concentrations during hospital stay [13].

During a long-term follow-up, asymptomatic post-COVID cohorts failed to show a clear cardiac involvement [37] while invasive measurement during exercise in patients with exertional dyspnea shows that the main exercise limitation regards peripheral oxygen extraction [14]. However, at six months of discharge, higher values of V̇_E_/V̇_CO2 slope_ have been linked to diminished HRR, suggesting that subjects with EV*in* may have cardiac autonomic dysfunction [26, 30, 39, 40]. An altered cardiac autonomic function may be one of the determinants of reduced peripheral extraction during exercise, and it is a general predictor of mortality in adults without a heart disease history [26]. Some studies confirm that normotensive post-COVID patients present a significantly higher blood pressure response in the post-exercise recovery, with an achieved lower O_2_ pulse at peak than controls without a history of COVID-19 [18]. The O_2_ pulse may have a non-specific interpretation due to its relationship with stroke volume and peripheral oxygen utilization. Recent data show that low levels of O_2_ pulse during exercise may be related to an increase in cardiovascular and all-cause mortality in some populations [15]. This leads to speculating that reduced O_2_ pulse peak values in COVID-19-recovered subjects could be a significant measure of health outcome. Low O_2_ pulse at peak is a consequence of a reduced V̇O_2peak_ during short-term follow-up. Already at 6 months of follow-up up to a year after hospital discharge for COVID-19, O_2_ pulse and V̇O_2peak_ increased significantly [1, 11]. In our longer follow-up, we document a significant global improvement of the O_2_ pulse from 6 to 34 months, despite no significant increase in VO_2peak_. The same was true for V̇_O2_/W_slope_, which generally increased as a sign of recovery of the hemodynamic response and the peripheral oxygen utilization. Of note, the selective increase of V̇_O2_/W_slope_ in pEV*in* patients has not a clear interpretation but may be related to the high variability of the few patients considered as pEV*in* group. In the context of the relationship between the cardiovascular response and the hyperventilation pattern, we demonstrate a significant correlation between the V̇_O2_/W_slope_, O_2_ pulse at peak, OUES and PET_CO2_ (Table 3), but only O_2_ pulse at peak and OUES with PET_CO2_ at rest.

Similarly to O_2_ pulse, OUES values represent an individual’s cardiorespiratory reserve and indicate how effectively oxygen is extracted and utilized by the body [25]. The prognostic potential of OUES has been examined in some clinical populations, such as patients with heart failure and very recently, the determination of OUES on healthy males has proved its prediction in all-cause mortality [17, 38]. Our data about the correlation between the hyperventilation and OUES, similarly for O_2_ pulse, define this variable as potentially prognostic for COVID survivors.

Data about the exercise training on parameters of cardiovascular response in patients with chronic obstructive pulmonary disease (COPD) report OUES - but also O_2_ pulse - as susceptible to changes, as a sign of an enhancement of ventilatory function upon exercise [39]. In the context of post-COVID patients, although in a single survivor patient from critical COVID-19 illness, and the data requires scientific confirmation, home-based exercise training has been demonstrated to produce a remarkable increment not only of V̇_O2_ peak but also of the OUES, with a consensual reduction in V̇_E_/V̇_CO2_ and exertional dyspnea [40].

Our study’s strength is related to evaluating the EV*in* for a very long time from COVID-19 discharge (pEV*in*). Although we report a small number of patients (an explicit limitation), this was related to a selective approach excluding patients having a condition potentially influencing the exercise ventilation assessment. We included a healthy population with normal exercise capacity and pulmonary function tests. This may also be considered a study strength because we excluded any potential cause of ventilatory inefficiency. Finally, we lack same-time data concerning the structural pulmonary (by thorax computed tomography scan) and cardiac (by echocardiography) damage. There is a possibility that these data could have confirmed a coexistent organic residual alteration.

In conclusion, our longitudinal data analysis on COVID-19 survivors, performed at 34 months from discharge, confirms the persistence of exercise ventilatory inefficiency in 16% of subjects. These subjects exhibit a hyperventilation status that correlates closely with an altered and unfavorable cardiovascular response to exercise. These observations underscore the importance of prolonged follow-up studies in individuals recovering from COVID-19.

### Electronic Supplementary Material

Below is the link to the electronic supplementary material.

Supplementary Material 1

## Data Availability

All data generated or analyzed during this study are included in this published article.
