# Supplementary material for: Persisting exercise ventilatory inefficiency in subjects recovering from COVID-19. Longitudinal data analysis 34 months post-discharge
Source: BMC Pulm Med. 2024 May 25;24:258. doi: 10.1186/s12890-024-03070-1 (PMC11128102; doi:10.1186/s12890-024-03070-1)
Supplement: Supplementary file 1 — Supplementary Material 1 [file 12890_2024_3070_MOESM1_ESM.docx]

Supplementary Table 1. General, functional and CPET-related variables comparing subjects with EV*in* at 6 months and pEV*in* at 34 months.

| Variables | Subjects with EV*in* (N=8) | Subjects with pEV*in* (N=5) |
| --- | --- | --- |
| Age, y | 56 [12] | 58 [13.1] |
| Male, n (%) | 7 (87) | 4 (80) |
| Current or former smokers, n (%) | 5 (62) | 3 (60) |
| Arterial hypertension^*^, n (%) | 2 (25) | 1 (20) |
| BMI, kg/m^2^ | 27.2 ± 3.1 | 27.3 ± 3.2 |
| FEV_1_, % predicted | 121.1 ± 14.4 | 126.2 ± 9.9 |
| FVC, % predicted | 122 [13] | 124.5 [18] |
| FEV_1_/FVC, % | 81.6 ± 4.3 | 82.3 ± 5.4 |
| TLC, % predicted | 100.7 ± 9.7 | 100.5 ± 13.2 |
| DL_CO_, % predicted | 93.8 ± 12.7 | 91.7 ± 17.5 |
| PaO_2_, mmHg | 98.6 ± 10.2 | 96.4 ± 12 |
| PaCO_2_, mmHg | 38.6 ± 3.3 | 39.6 ± 3 |
| pH | 7.42 ± 0.02 | 7.42 ± 0.01 |
| 6MWT, total distance walked meters | 591.4 ± 100.6 | 562.8 ± 101 |
| mMRC, score | 1 [1] | 1 [1] |
| IPAQ (inactive/minimally active/  HEPA active), n (%) | 5(62)/3(37)/0(0) | 3 (60)/1(20)/1(20) |
| METs, total | 871 [1791] | 1386 [2068] |
| Workload, watts | 170.6 ± 55.8 | 148.8 ± 43.4 |
| V̇_O2_ at peak, ml | 2223 ± 538 | 1960.2 ± 493.7 |
| V̇_O2_ at peak, ml/kg/min | 27.6 ± 7.8 | 24.7 ± 7.2 |
| V̇_O2_ at peak, % predicted | 99.5 ± 17.5 | 91.6 ± 6.1 |
| V̇_O2_/W_slope_ | 9.32 ± 1.37 | 9.18 ± 1.75 |
| V̇_E_/V̇_CO2_ _slope_ | 33.7 ± 1.4 | 33.9 ± 1.6 |
| V̇_E_/V̇_CO2_ _nadir_ | 29.7 ± 2.5 | 30.5 ± 2 |
| V̇_E_/V̇_CO2_ at θ_L_ | 31 ± 1.4 | 31 ± 1.7 |
| V̇_E_/V̇_CO2_ _intercept_ | -0.58 ± 3.4 | -0.24 ± 3.6 |
| V̇_E_ at rest, L/min | 17.8 ± 6.5 | 17.6 ± 7.2 |
| V̇_E_ at peak, L/min | 94.3 [27.2] | 101.3 [38.6] |
| RR change^§^, breath/min | 21.5 ± 3.2 | 22.2 ± 4.1 |
| O_2_ pulse at peak, mL/bpm | 14.4 ± 3.1 | 12.6 ± 2.3 |
| OUES, L/min | 1.11 ± 0.25 | 0.98 ± 0.20 |
| HR_max_ | 153 ± 13.1 | 153.6 ± 13.9 |
| HRR, beats/minute | 22 ± 6.9 | 20.6 ± 6 |
| HR/V̇_O2_ slope, L^-1^ | 52.8 [55.6] | 77.5 [56] |
| Perceived peak dyspnea^#^ | 17 [5] | 17 [3.5] |
| Perceived peak fatigue^#^ | 18 [2] | 18 [3] |
| Length of hospital stay, days | 6.5 [6] | 6 [11] |
| Needing of oxygen therapy, n (%) | 6 (75) | 4 (80) |
| Needing of ventilatory support, n (%) | 3 (37) | 2 (40) |
| Needing of ICU admission, n (%) | 2 (25) | 2 (40) |
| Pulmonary embolism, n (%) | 0 (0) | 0 (0) |
| PaO_2_/FiO_2_ at admission (n=16) | 323.8 ± 66.1 | 307.9 ± 84.2 |
| PaO_2_/FiO_2_ <300, n (%) | 1 (13) | 1 (50) |
| PaCO_2_ at admission (n=16) | 36.7 ± 9.4 | 35 ± 12.7 |

Data are shown as the number of subjects (%), means ± SD or medians [IQR-interquartile range]. In bold are reported significant values.

^*^Subjects with arterial hypertension were treated with ACE inhibitors (N=6, 19%), β-blockers (N=4, 12%), and Ca^2+^ antagonist (N=3, 9%); ^§^Calculated as value at peak less value at rest; ^#^Described as a Borg 6-20 perceived exertion rate score.

*Abbreviations*: pEV*in*, persisting exercise ventilatory inefficiency; BMI body mass index; FEV_1_, forced expiratory volume at 1^st^ second; FVC, forced vital capacity; TLC, total lung capacity; DL_CO_, diffusion capacity for carbon monoxide; PaO_2_, partial arterial oxygen pressure; PaCO_2_, partial pressure of arterial carbon dioxide; 6MWT, six-minute walking test; mMRC, modified Medical Research Council dyspnea score; IPAQ, international physical activity questionnaire; HEPA, health-enhancing physical activity; METs, metabolic equivalent of task; V̇_O2_, oxygen uptake; V̇_E_/V̇_CO2 slope_, the slope of V̇_E_ to carbon dioxide output-V̇_CO2_ ratio; θ_L_, the first ventilatory threshold; V̇_E_/V̇_CO2 intercept_, point of intercept of V̇_E_ to carbon dioxide output-V̇_CO2_ ratio; V̇_E_, minute ventilation; RR, respiratory rate; OUES, oxygen uptake efficiency slope; HRR, heart rate recovery; ICU, intensive care unit.
